# Comparative Review of the Conserved UL24 Protein Family in Herpesviruses

**DOI:** 10.3390/ijms252011268

**Published:** 2024-10-19

**Authors:** Odelia Orbaum-Harel, Ronit Sarid

**Affiliations:** 1 The Mina and Everard Goodman Faculty of Life Sciences, Bar-Ilan University, Ramat Gan 5290002, Israel; del.orbaum@gmail.com; 2 Advanced Materials and Nanotechnology Institute, Bar-Ilan University, Ramat Gan 5290002, Israel

**Keywords:** UL24, herpesvirus, open reading frame 20 (ORF20), UL76, ORF35

## Abstract

The UL24 protein family, conserved across all subfamilies of Orthoherpesviridae, plays diverse and significant roles in viral replication, host–virus interactions and pathogenesis. Understanding the molecular mechanisms and interactions of UL24 proteins is key to unraveling the complex interplay between herpesviruses and their hosts. This review provides a comparative and comprehensive overview of current knowledge on UL24 family members, including their conservation, expression patterns, cellular localization, and functional roles upon their expression and during viral infection, highlighting their significance in herpesvirus biology and their potential functions.

## 1. Introduction

The Orthoherpesviridae family encompasses over 100 viral species of considerable veterinary and medical importance [1]. Members of the family feature enveloped virions that package a double-stranded DNA genome within a capsid surrounded by a thick tegument layer. A distinctive and universal characteristic of this family is the establishment of latency following initial infection, which can manifest as either symptomatic or asymptomatic. Based on broad biological criteria, including cell tropism and characteristics of the infection cycle, members of the Orthoherpesviridae are classified into three subfamilies: Alpha, Beta and Gammaherpesvirinae [2].

The genome of Orthoherpesviridae ranges from 125–241 kbp, exhibiting a variable number of open reading frames (ORFs) within the range of 70 to 170, with a subset of at least 26 protein-coding genes conserved across all family members [3]. Conserved genes encode capsid proteins, components of the DNA replication and packaging machinery, nucleotide-modifying enzymes, tegument proteins, envelope glycoproteins, and, to a lesser extent, regulatory proteins [3]. This conservation reinforces the notion that, despite genetic and biological diversities among Orthoherpesviridae members, they share common features in various aspects of their infection cycle, and the conserved genes are likely to share common roles in the biology of these viruses [2,3].

The UL24 protein family, the primary focus of the present review, is conserved among all three subfamilies of the Orthoherpesviridae, suggesting a common ancestral gene dating to approximately 180–220 million years ago prior to the emergence of the three subfamilies [4,5]. A UL24 amino acid sequence and structural alignments study revealed sequence similarity across herpesviruses, consistent preservation of hydrophobicity and topology patterns, and almost complete conservation of a PD-(D/E)XK signature motif providing a putative active site for endonuclease activity [6]. Yet, the overall understanding of the function of the UL24 protein family is limited, and its exact function during infection is not entirely clear. Insights gained on several UL24 family members, however, provide valuable perspectives into their potential roles in viral pathogenesis and host–virus interactions. Given the limited knowledge surrounding UL24 protein family members, there is a compelling need for further investigation into their structure, function, and molecular interactions. This review aims to provide a comprehensive overview of the UL24 protein family. By summarizing the existing literature, we intend to illuminate the current understanding of these proteins and underscore the gaps in our knowledge with the ultimate goal of stimulating further research into this intriguing protein family.

## 2. UL24 Family Gene Cluster

The UL24 family members of the alpha and gammaherpesvirinae are situated within a conserved gene cluster that encodes UL22 (glycoprotein H, gH), UL23 (Thymidine kinase, TK), UL24 and UL25 (capsid vertex component, CVC). The UL24 gene is bounded on one side in a head-to-head orientation by the TK gene and on the other side by the CVC gene, which is encoded on the same strand (Table 1). The flanking genes occasionally overlap with the UL24 ORF. This genomic organization is conserved across the human herpesviruses including Herpes simplex virus type 1 (HSV-1) and type 2 (HSV-2), Varicella Zoster virus (VZV), Epstein–Barr virus (EBV), and Kaposi’s sarcoma-associated herpesvirus (KSHV), as well as animal herpesviruses such as the Pseudorabies virus (PRV), Bovine herpesvirus 1 (BHV-1), Duck enteritis virus (DEV), Equine herpesvirus 1 (EHV-1), Testudinid herpesvirus 3 (TEHV-3), and murine gammaherpesvirus 68 (MHV-68). Similarly, betaherpesvirinae, including the human cytomegalovirus (hCMV), human herpesvirus 6A/B (HHV-6A/B) and human herpesvirus 7 (HHV-7), encode a CVC protein upstream of UL24 on the same strand. However, as they lack the TK gene, they encode gH downstream on the complement strand [2].

## 3. UL24 Family Expression Kinetics

The HSV-1 UL24 30 kDa protein can potentially be translated from two distinct polycistronic sets of transcripts: short transcripts of 1.2 and 1.4 kb, processed at the same UL24 polyadenylation site, and long transcripts of 5.6 and 5.4 kb, processed at the UL26 polyadenylation site. These transcripts differ in their expression levels and exhibit, respectively, early and leaky-late lytic kinetics [7,8]. However, the late accumulation of the UL24 protein and its decreased expression, though not complete elimination, in the presence of the viral DNA polymerase inhibitor phosphonoacetic acid (PAA) suggest that this protein is expressed with leaky-late or late-lytic kinetics [7,8,9]. Similarly, PRV UL24 has been classified as a late gene based on its expression kinetics, PAA sensitivity, and clustering analysis of co-regulated genes [10]. Furthermore, kinetics analysis, as well as utilization of a mutated HSV-1 containing a deletion that removes the promoter and the start site of the longest 5.6 kb transcript, which originates at an upstream start site, suggest that this long transcript serves as the primary source of UL24 expression [11]. A third set of transcripts of 5.2 and 0.9 kb originates from a transcription start within UL24 ORF and was suggested to encode a truncated 18 kDa UL24 protein that starts at the third in-frame ATG site at amino acid 122 within UL24 referred to as UL24.5. Like UL24, the UL24.5 protein is also expressed with late kinetics. Of note, this potential Methionine initiation codon is conserved across HSV-1, HSV-2 and VZV, yet it is absent in beta and gammaherpesviruses [12].

Expression of the betaherpesvirus hCMV pUL76, represented by a 38 kDa protein, has been detected in hCMV-infected cells two hours post-infection, peaked at 24 h, and remained throughout the late reproductive cycle of hCMV infection [13]. Later studies identified two UL76 transcripts classified as late based on their expression kinetics and sensitivity to cycloheximide and PAA [14,15]. Further investigation aiming to explain the presence of pUL76 shortly after an infection, however, revealed that mature viral particles contain monomeric UL76 in the soluble envelope fraction and a high-molecular-mass UL76 protein (H-UL76), composed of disulfide cross-linked UL76 homo- or hetero-oligomers, in the insoluble tegument/capsid fraction, that can be transported into cells during infection [14]. This finding aligns with HSV-2 UL24 protein, which has been shown to be associated with purified virions and intracellular C-capsids [9], and with MHV-68 ORF20, which was found in purified extracellular virions [16]. Nevertheless, other studies did not detect UL76 [17,18], UL24 [11,19], and KSHV ORF20 [20,21] in virions, suggesting that their incorporation into virions may be cell-type specific or occur in residual quantities. Of note, UL76 is identified as one of the viral latency-associated transcripts in CD34+ hematopoietic cells latently infected with hCMV, specifically in the CD34+ CD38- subpopulation, which maintains the latent hCMV genome and supports viral reactivation [22]. However, contradictory results show that UL76 transcripts are not detected in latently infected myeloid progenitor cells [23], a discrepancy that needs to be resolved.

Kinetic profiling of gammaherpesvirus KSHV protein expression in endothelial cells has classified ORF20 as a late gene product. It shares the same cluster with viral structural components whose expression increases 48–72 h following lytic reactivation and shows marked sensitivity to PAA [24]. This finding aligns with a previous study in which ORF20 exhibited late expression kinetics and sensitivity to the viral DNA replication inhibitor cidofovir in KSHV-infected primary effusion cells [25], as well as our observation in iSLK cells [26]. Similarly, MHV-68 ORF20 has been described as exhibiting an early-late lytic expression kinetic [27]. Notably, ribosome profiling of KSHV-infected iSLK cells, in which viral lytic replication was induced combined with in silico prediction, identified three colinear isoforms of KSHV ORF20 protein consisting of 320, 297 and 257 amino acids (aa). All isoforms share a termination codon, while two initiate translation with a Methionine start codon, resulting in either a full-length protein (ORF20-FL) or a 64-aa truncated short isoform (ORF20-S) and an intermediate isoform (ORF20-IM) that uses a non-canonical CTG start codon, encoding Leucine at position 64 [28].

## 4. Protein Conservation, Motifs and Structure

Understanding the shared characteristics of a protein family’s amino acid sequence and structure can provide a foundational framework for comprehensive research. UL24 family members comprise highly basic amino acid sequences. Multiple sequence alignments of UL24 family proteins reveal five conserved homology domains (HD-1 to HD-5) [6,26,29,30]. Notably, the homology of the KSHV ORF20 protein is predominantly observed in the short isoform, with the 64 N-terminal amino acids not being conserved among UL24 proteins. A predicted bipartite nuclear localization signal (NLS) within the HSV-1 UL24 C-terminus was found nonessential for its nuclear localization, yet the initial 60 aa of HSV-1 UL24 does contain a nucleolar-targeting signal [31]. In addition, a possible “non-classical” nuclear export signal (NES) between aa 250 and 258, containing five hydrophobic residues, has been reported capable of directing UL24 export from the nucleus to the cytoplasm [32]. Similarly, hCMV UL76 is predicted to contain six NLS motifs [33], whereas KSHV ORF20 encodes potential nuclear and nucleolar localization sequences within its carboxy terminus, yet these sequences were not exclusively required for ORF20 localization [34].

**Figure 1:**
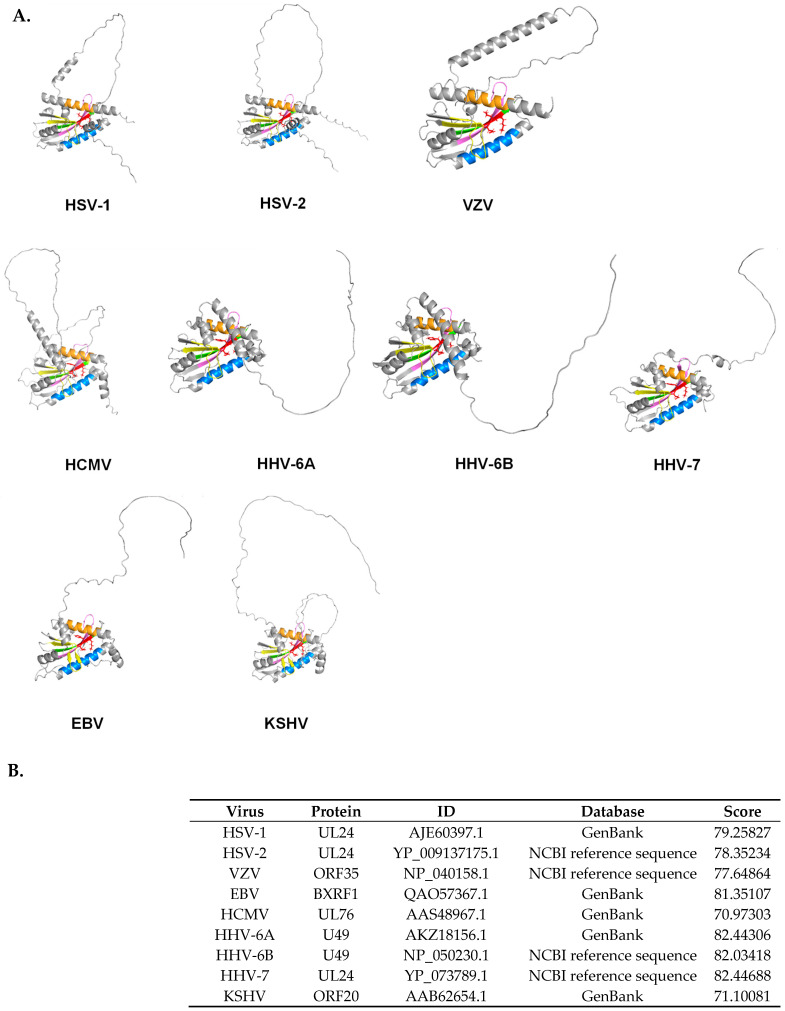
Predicted structures of proteins from the UL24 family across nine human herpesviruses, generated using the AlphaFold2 AI system. (**A**) Cartoon models of the predicted structures for HSV-1 UL24, HSV-2 UL24, VZV ORF35, HCMV UL76, HHV-6A U49, HHV-6B U49, HHV-7 UL24, EBV BXRF1, and KSHV ORF20 are shown from the same angle. The homology domains (HDs) are color-coded as follows: HD-1—orange, HD-2—yellow, HD-3—green, HD-4—blue, and HD-5—pink. The putative endonuclease motif is highlighted in red as a stick model. (**B**) A table summarizing the local distance difference test (pLDDT) confidence scores.

Using the Meta-BASIC tool [35], which is known for its sensitive fold recognition through comparing sequence profiles with anticipated secondary structure predictions, UL24 family proteins have been predicted to encode a PD-(D/E)XK endonuclease motif belonging to a superfamily of restriction endonuclease-like proteins [6]. Enzymes of this class play crucial roles in vital biological processes such as safeguarding against foreign DNA, DNA damage repair, resolution of Holliday junctions, and DNA recombination. Their common core structure, characterized by an αβββαβ topology, consists of a mixed four-stranded β-sheet flanked by α-helices on either side, with the PD-(D/E)XK motif serving as a distinctive identifier of the active site residues responsible for cleaving diverse nucleic acid substrates [6]. As almost all UL24 homologs carry a common core topology and exhibit complete conservation of the PD-(D/E)XK signature within HD-3, this predicted motif is expected to play a fundamental role in the function of the UL24 protein family. To construct a three-dimensional structural model of the UL24 protein family, we used the AI-based algorithm AlphaFold2 [36] and obtained the predicted structures. As shown in Figure 1, the structures of human herpesvirus UL24 family proteins revealed similar features. Notably, all homologs possess a number of alpha-helical domains, with the conserved ELK motif consistently residing within a beta-sheet, deeply embedded in the protein core. Interestingly, when generating and rotating 3D models, we observed an apparent channel containing the ELK motif. This structural feature may suggest an enzymatic function for these proteins, with the channel potentially serving as a conduit to the ELK residues, which could play a catalytic role. The AlphaFold2 predictions were supported by high-confidence algorithm scores, further reinforcing the potential enzymatic function of the UL24 protein family (Figure 1B). This prediction has yet, however, to be supported by functional assays demonstrating the endonuclease activity of these proteins.

## 5. Cellular Localization of UL24 Family Members

Understanding the cellular distribution and localization of viral proteins during ectopic expression and various stages of infection is important for unraveling their functions, interactions, and potential impact on host cellular processes. Nuclear localization characterizes most UL24 homologs, while a subset displays nucleolar localization, highlighting a specific affinity for nucleolar regions and suggesting potential interactions with nucleolar components. Biochemical fractionation has shown that in HSV-1-infected Vero cells, UL24 increasingly localizes to the nuclear fraction over time, with approximately 70% of UL24 in the nucleus by 15 h post-infection [11]. Furthermore, immunofluorescence assays with Vero cells infected with a recombinant virus expressing C-terminal HA-tagged HSV-1 UL24 detected UL24 in both the nucleus and cytoplasm, with notable nucleolar accumulation at 9 h post-infection and a prominent perinuclear localization at later time points. Notably, UL24 was excluded from Cajal bodies and viral replication compartments, detected by their corresponding markers, and did not colocalize with Coilin and ICP8 [37]. Transient expression of UL24 resulted in both nuclear and Golgi localization, with most cells exhibiting diffuse nuclear staining and a minority showing a nucleolar staining pattern [31,38]. None of the five HDs was essential for UL24 localization to the Golgi apparatus and nucleolus [31].

Consistently, when HSV-2 UL24 was expressed alone in Vero cells, it predominantly localized to the nucleus, with lighter cytoplasmic and nucleolar staining at 24 and 48 h post-transfection [9]. A nucleolar targeting signal was identified within the first 60 aa of UL24, while the C-terminal domain (aa 190–269) was excluded from the nucleus and accumulated in the Golgi apparatus [31]. A recent study using expression plasmids with nested deletions within the C-terminal domain of HSV-1 UL24 revealed nuclear staining for several UL24-deleted forms. Particularly, mutations targeting a predicted NES significantly increased nuclear localization, suggesting that UL24 can shuttle between the nucleus and cytoplasm. Replacing a threonine phosphorylation site had no discernible impact on UL24 localization. Treatment with the nuclear export receptor CRM-1-specific inhibitor leptomycin B impeded the nuclear export of UL24 in transfected cells, indicating that ectopically expressed UL24 shuttles between the nucleus and the cytoplasm in a CRM-1-dependent manner. NES-dependent shuttling of UL24 was also observed during infection; however, it was CRM-1 independent, suggesting the involvement of an alternative or additional export pathway mediated by other export receptors. Nevertheless, recombinant viruses harboring substitutions in the NES region of UL24 resulted in the accumulation of UL24 within the nucleus [32]. Similarly, UL24 encoded by PRV [39] and EGFP-tagged pORF35 of VZV were predominantly detected in the nuclei of transfected cells, irrespective of the tag position, while a recombinant VZV encoding flag-tagged ORF35 expressed this gene product in the nuclei of infected cells at 24 and 96 h post-infection [40]. The UL24 gene product of the DEV presents different cellular localizations in different cells during transfection and infection. When transiently expressed, it is localized in the nucleus and cytoplasm in various proportions. During infection, however, it is in the perinuclear region regardless of infection time-point, with little protein in the nucleus. This has been attributed to the interaction of UL24 with UL54, which enables UL24 to shuttle from the nucleus to the cytoplasm during infection [41,42]. Furthermore, if HSV-1 UL24 interacts with UL54, as observed with DEV UL24, this interaction may facilitate the nuclear export of UL24. These observations indicate that UL24 possesses several targeting signals that govern its intracellular distribution and trafficking.

Similar to alphaherpesviruses, UL76 encoded by the betaherpesvirus hCMV was initially shown to distribute in globular foci within the nuclei, likely representing the nucleolar fraction [13]. Later, it was found to predominantly localize to the nucleus and nucleolus, significantly reducing the number of promyelocytic leukemia (PML) bodies [38]. Transiently expressed UL76 mutants containing aa 1-190 and aa 187-325 fused to EGFP revealed nuclear fluorescence and diffuse distribution throughout most of the cell, respectively [33]. In the context of the hCMV infection, UL76 predominantly manifested as globular nuclear aggresomes, possibly representing nucleoli. Live cell monitoring unveiled a dynamic behavior where UL76 protein was soluble in the nucleoplasm but transitioned to an insoluble form in the aggresomes, suggesting potential initiation of conformational changes in the nucleoplasm [33].

An initial screen of the gammaherpesvirus KSHV protein localization identified ORF20 in the nucleus [43]. Further analysis, using immunofluorescence (IF) and confocal microscopy to elucidate the subcellular distribution of ORF20 isoforms within different transfected cell types, including during lytic reactivation, detected a predominant nuclear and nucleolar localization, demonstrating colocalization with the nucleolar protein Fibrillarin [34]. This study also revealed a similar distribution of transfected myc-tagged ORF20 encoded by MHV-68, as well as its homologs HSV-1 UL24, hCMV, and mCMV UL76, which colocalized with Fibrillarin in HeLa cells [29], while an EBV protein localization screen identified BXRF1 in the nuclear and nucleolus-like compartment [44].

## 6. Functional Studies of UL24 Family Members

Elucidating the functions of individual proteins within a protein family can advance our understanding and guide further research into their shared and intricate roles in diverse biological processes. Accordingly, substantial research efforts have been dedicated to exploring members of the UL24 family, with each study providing potential insights into the functions of other family members, thereby expanding our collective understanding. This section reviews these studies, highlighting multiple functions within and outside the context of viral infection.

### 6.1. Cell Viability and Cell Cycle Progression

Proteins of the UL24 family have been identified as having dynamic effects on cell cycle progression and apoptosis. This pattern has been observed in several proteins within the UL24 family, including HSV-1 UL24, hCMV UL76, MHV-68, and KSHV ORF20, which have been implicated in instigating G2 cell-cycle arrest and apoptosis in mouse and human cells [45,46]. Initial observations show that expression of MHV-68 ORF20 blocks cell progression into mitosis by inactivating the Cdc2-Cyclin B complex, thereby arresting cells at the G2 stage of the cell cycle and leading to apoptotic cell death. These findings, illustrating predominantly inactive phosphorylated Cdc2 complexed with Cyclin B, indicate the virus protein’s ability to impede cell progression into mitosis by targeting the Cdc2–cyclin B complex [45]. A subsequent study by the same group revealed similar characteristics in cells transduced with recombinant lentiviruses expressing HSV-1, hCMV and KSHV UL24 homologs [46]. Intriguingly, however, the absence of observable apoptosis within the initial 2-day post-transduction period, as evident in a TUNEL assay, raises questions about the relevance of the putative UL24 endonuclease activity to the observed phenotype, given that endonuclease activity was expected to predominantly manifest shortly after expression [46]. Of note, DNA fragmentation and formation of micronucleus were also evident upon expression of DEV UL24 [41]. An additional study demonstrated the colocalization of EGFP-UL76 and EGFP-UL76C (aa 187-325) with phosphorylated histone γ-H2AX, which was rare in cells transfected with pEGFP or pEGFP-UL76N (aa 1-190) [47]. In line with this, cells stably transfected with hCMV UL76 accumulated chromosomal aberrations, characterized by micronuclei and misaligned, lagging, and bridging chromosomes during mitosis, along with elevated occurrence of aberrant spindles and enhanced nuclear foci containing γ-H2AX [48]. Although a marginal increase in cells with supernumerary centrosomes was evident, the central role of UL76 in promoting DNA damage signal activation through γ-H2AX and ensuing foci formation cannot be overlooked. Notably, the degree of DNA breaks directly correlates with UL76 protein levels, underscoring its role in chromosomal abnormalities. Whether UL76 induces these abnormalities or whether cells expressing UL76 are unable to repair DNA damage remains to be elucidated. Nevertheless, the expression of UL76 was shown to induce ATM-dependent activation of DNA damage, leading to the phosphorylation of p53 and H2AX, which up-regulated the nuclear factor kappa B (NF-ĸB) pathway and promoted transcription of interleukin 8 (IL-8), supporting the replication of hCMV. This effect was moderately reduced upon mutating the putative endonuclease motif of UL76, indicating that while this motif contributes to IL-8 transcription, it is not essential to it [49]. Within this context, it is interesting to note that UL76 directly binds to the BRCT domain of PARP-1, reducing PARP-1 PARylation levels and possibly the PARylation of other substrates, such as histones. This function may avoid overactivation of PARP-1 during infection, thereby controlling NAD+ levels and preventing cell death. However, PARP-1 inhibition can result in DNA damage and abnormal mitosis. Nevertheless, UL76 was shown to accumulate at DNA damage sites through its binding to poly (ADP-ribose) (PAR) chains. Notably, HSV-1 UL24 and KSHV ORF20 do not bind PARP-1 and PAR chains, suggesting that this interaction is unique to UL76. Its exact role during infection and its association with DNA damage response in the context of infection require further exploration [50].

### 6.2. Regulation of Gene Expression

Investigations involving co-transfection experiments have unveiled a distinct correlation between HSV-1 UL24 expression and reduction in the expression of several viral proteins and transcripts [51]. Co-expression of UL24 with the large HSV-1 ribonucleotide reductase (R1) subunit, the small ribonucleotide reductase (R2) subunit, infected cell protein 27 (ICP27) or thymidine kinase (TK) led to reduced proteins and transcripts levels in both non-human primate and human cells. This effect appears to be mediated through the coding region, although the involvement of regulatory elements cannot be ruled out. Interestingly, substitution mutations G121A and E99A/K101A, targeting conserved residues within HSV-1 UL24, hindered this regulatory function, demonstrating the importance of these residues for UL24’s regulatory function [51]. Similarly, UL24 from Herpes B virus (BV) also reduced R1 expression in transfection experiments [51].

Ectopic expression of HSV-1 UL24 has also been demonstrated to substantially inhibit cGAS-STING-mediated promoter activation of interferon (IFN) and IL-6. This inhibition was also evident during infection, showing that the cGAS-STING-mediated DNA-sensing signaling pathway was suppressed by UL24. Mechanistically, HSV-1 UL24 selectively blocks the NF-κB signaling pathway while sparing interferon-regulatory factor 3 (IRF3) promoter activation [52]. Curiously, this function contrasts with its homolog UL76, which activates the NF-ĸB pathway [49]. This unique effect involves the interaction of UL24 with p65 and p50, reducing their nuclear translocation, which is prompted by tumor necrosis factor alpha (TNF-α). Mutational analysis has pinpointed the region spanning aa 74 to 134 of HSV-1 UL24 as the crucial determinant for inhibiting cGAS-STING-mediated NF-κB promoter activity, whereas the conserved endonuclease motif was dispensable. This insight marks the first report of UL24’s pivotal involvement in the evasion of the antiviral response during HSV-1 infection [52]. Similarly, ectopic expression of PRV UL24 effectively thwarts TNF-α-mediated NF-κB activation. However, the suppression of NF-ĸB by PRV UL24 involves targeting p65 for degradation via the proteasome pathway while sparing p50. This was associated with significant downregulation of its targets IL-6 and IL-8. In line, relatively higher activation of NF-ĸB reporter gene expression was evident in cells infected with mutated PRV that failed to express UL24 compared with those infected with wild-type PRV [39].

Furthermore, PRV UL24 inhibited IFN-β activation induced by poly(dA:dT) or stimulated by cGAS-STING. This inhibition, however, was attributed to an interaction between UL24 and interferon regulatory factor 7 (IRF7), which mediates the proteasomal degradation of IRF7 [53]. Of note, the UL24 homolog encoded by DEV alphaherpesvirus has also been identified as a significant inhibitor of the cGAS-STING-mediated activation of the IFN-β promoter by promoting the degradation of IRF7 through the ubiquitin-proteasome pathway following transfection in duck embryo fibroblasts [54,55]. Finally, infection with the PRV UL24-deficient virus resulted in enhanced levels of the innate immune response gene zinc finger CCHC-type containing protein 3 (ZCCHC3) gene product, thus supporting the role of UL24 in evading antiviral response [56].

Regarding betaherpesvirinae, a screen for hCMV proteins that promote expression from the major immediate-early promoter/enhancer (MIEP) identified UL76 as a regulatory protein that modulates gene expression in a dual manner, either activating or repressing depending on its dose and the target promoter/enhancer. This study did not, however, explore the underlying mechanism behind this observation [13]. Furthermore, the constitutive expression of the UL76 protein down-regulates early viral protein expression during infection [57]. A later study identified UL76 as a key factor in inducing expression of the inflammatory chemokine IL-8 in a dose-dependent manner as a result of DNA damage response [49]. The up-regulation of IL-8 was found to involve activation of the NF-κB signaling pathway, whereas inhibition of IKK-β or degradation of IκBα abolished activation of the NF-ĸB pathway and its binding to the IL-8 promoter. Additionally, the UL76-mediated induction of IL-8 requires the ATM kinase and correlates with the phosphorylation of NEMO on serine 85, indicating that UL76 activates the NF-ĸB pathway via the DNA damage response, similar to the effects of genotoxic drugs. Importantly, a UL76 deletion mutant virus is significantly less efficient at stimulating IL-8 production than the wild-type virus. There is also a significant reduction in IL-8 secretion when ATM -/- cells are infected with wild-type hCMV, indicating that ATM is involved in hCMV-induced IL-8 production. Of note, the levels of IL-8 secreted by cells expressing a mutant UL76 gene within three putative endonuclease signature amino acids were reduced compared with those expressing the wild-type UL76 gene, although they remained significantly higher than those from cells containing the control vector. These results indicate that the putative endonuclease activity is not essential for the induction of IL-8 [49]. Finally, UL76 was shown to significantly down-regulate the expression of its overlapping essential viral replication protein UL77, possibly via the regulation of the translation re-initiation mechanism. This was demonstrated using transfection of expression plasmids and infection with recombinant viruses containing selected mutations within UL76. This may enable UL76 to control the temporal expression and levels of UL77, thereby optimizing conditions for efficient viral replication [57].

### 6.3. Cellular Protein Localization

The first indication of involvement of UL24 homologs in the dispersion of nucleolar proteins was obtained by comparing infection with the wild-type HSV-1 and mutant viruses that do not express UL24 [37]. In line with previous reports [58,59], this study observed the distribution of the nucleolar proteins Fibrillarin and Nucleolin during infection. However, the distribution of Nucleolin was reduced only when cells were infected with the UL24-deficient viruses, suggesting that its dispersion depends on UL24 but not that of Fibrillarin [37]. The dispersal of other nucleolar proteins, including the transcription factor UBF and the RNA polymerase I catalytic subunit, RPA194, was also evident during HSV-1 infection but was found to be independent of UL24 [60]. It was subsequently shown that UL24 is sufficient to induce dispersal of Nucleolin throughout the nucleus in the absence of infection. The conserved N-terminal domain of UL24 was sufficient for Nucleolin dispersal, and all conserved homology domains (HDs 1-5) were required for this effect [31]. An examination of Nucleolin distribution with a panel of single and double UL24 substitution mutations within the most conserved amino acid residues revealed that transfection with UL24 mutants exhibiting wild-type distribution correlated with high Nucleolin dispersal levels. Mutations within specific amino acids, including those in the first HD and the PD-(D/E)XK putative endonuclease motif, have the most pronounced effects on Nucleolin dispersal. Among them, the E99A/K101A mutation exhibited the most notable impact on nucleoli modification, leading to a decreased ability to induce Nucleolin dispersal. This was also seen in infections with an HSV-1 mutant encoding the E99A/K101A UL24, similar to the UL24-deficient virus [61].

The dispersion of another nucleolar protein, B23, also termed Nucleophosmin 1(NPM1), during HSV-1 infection was also contingent on UL24, specifically on its conserved N-terminal domain. Similar to Nucleolin dispersion, the mutational analysis highlighted the importance of UL24’s endonuclease motif in B23 dispersal in both transfected and infected cells [62], suggesting they share the same mechanism of protein dispersal. Nevertheless, it is unclear whether this effect is directly or indirectly mediated by UL24, and an interaction between these nucleolar proteins and UL24 has not been demonstrated. Intriguingly, the expression of ectopically introduced UL24.5 did not replicate the nucleolar protein dispersal effects observed for UL24 [12].

The effects of UL24 family proteins on cellular protein localization, both during ectopic expression and viral infection in viruses from the beta and gamma subfamilies of Herpesviridae, have not yet been investigated.

### 6.4. Distribution of Viral Glycoproteins

As UL24 deficiency results in a syncytial plaque phenotype [29,63], it has been suggested that UL24 affects the localization of viral proteins, potentially by influencing the organization of the ER and Golgi apparatus. Infecting human foreskin fibroblasts (HFFs) with the UL-24 null virus (UL24X) produced an altered Golgi structure characterized by reduced fragmentation and large networks of reticular structures. This extended cell-type-specific Golgi network was not, however, essential for syncytia formation; instead, it seemed to form as a consequence of syncytia development. Unlike wild-type-infected cells where viral glycoproteins B (gB), gD, gH, and gL are detected as extended blotches throughout the cytoplasm with limited nuclear membrane staining, in the UL24X-infected cells, these glycoproteins appeared as long, thin streaks running across the cell. Moreover, there was a notable decrease in the colocalized staining of gB and gD with F-actin at later stages in UL24X-infected HFFs [64]. Interestingly, the incorporation of gD into the viral envelope is significantly enhanced in virions produced upon infection with HSV-1 containing the UL24:C103Y substitution mutation [65].

### 6.5. Evasion of Antiviral Response

As detailed above, HSV-1 and PRV UL24 inhibit the promoter activation of IL-6, IFN, and IL-8 through the reduction of the DNA sensing pathway of cGAS-STING and the inhibition of the NF-ĸB pathway [39,52]. Further studies have shown a reduction in IFN-β, IκBα, MxA, and ISG20 mRNAs in the presence of UL24 and elevated expression of these genes in cells infected with UL24-null viruses [66,67]. Domain-mapping analysis pinpointed the N-terminal region (aa 1-90) of PRV UL24 as responsible for the effect on ISG20 transcription [67]. Additionally, PRV UL24 markedly inhibited the transcription of the antiviral interferon-stimulated gene oligoadenylate synthetase-like (OASL), which in turn reduced RIG-1 transcription and signaling that mediate the IFN response during PRV infection, thus indicating a critical role for UL24 in antagonizing host antiviral response [66]. Notably, in contrast to PRV UL24, KSHV ORF20 enhanced the RIG-1-induced expression of OASL in an IRF3-dependent but IFN-independent mechanism, leading to increased KSHV infection in an ORF20-dependent manner [34]. Increased viral replication by ORF20 was also evident with MHV-68, which does not interfere with the OASL and RIG-1 interaction and coimmunoprecipitates with OASL and RIG-1 through OASL binding. Furthermore, all potential ORF20 isoforms, as well as its homologs MCMV M76, hCMV UL76, and to a lesser extent HSV-1 UL24, co-immunoprecipitated with OASL. This interaction occurred independently of RNA-binding and the ubiquitin-like domain of OASL. Further examination revealed that KSHV ORF20 and OASL share nucleolar localization, a number of nucleolar and ribosomal interacting protein partners, and were individually copurified with the 60S and 40S ribosomal subunits, and when copurified, they were associated with polysomes [34]. However, ORF20 did affect the global translation rate, suggesting it may control the translation of a subset of mRNAs.

### 6.6. Protein Interaction

Beyond the functions detailed here, a number of potential interacting partners of KSHV ORF20 have been identified through affinity purification of ectopically expressed ORF20 in HEK-293 and HeLa cells coupled to mass spectrometry [34,68]. This includes the coiled-coil domain-containing protein 86 (CCDC86), whose depletion has recently been associated with chromosome organization, Ki-68 and Nucleolin localization, cytoplasmic aggregation and increased apoptosis, suggesting its involvement in chromosome segregation [68,69]. Interestingly, this phenotype overlaps the characteristics of cells that overexpress UL24 members. In addition, interactions with a large number of 40S and 60S ribosomal subunit proteins, as well as with LTV1 homolog involved in ribosome biogenesis, apoptosis-inducing factor 1 (AIF-1) and PRPF6 and PRP31 associated with spliceosome assembly, have been identified [34]. A more recent study utilized a biotin ligase proximity-labeling method to identify the proximal interactome of KSHV ORF20 during lytic reactivation. This revealed a number of protein partners, including ribosomal proteins and the KSHV DNA processivity factor ORF59, which was suggested to colocalize with ORF20 in viral replication compartments [70].

pUL76 was shown to interact with pUL31 both in the presence and absence of infection, resulting in an altered distribution of pUL31 within subnuclear domains [71]. Similar to pUL31, pUL76 was also found to reduce pre-rRNA levels in the absence of infection; however, when expressed with UL31, no further reduction in pre-rRNA levels was observed [71]. Of note is that pUL31 was later described as an inhibitor of DNA sensing by cGAS, yet the mechanism involved has not been revealed. Given the ability of UL24 homologs to reduce cGAS signaling, it is possible that this function could be mediated cooperatively [72]. In addition, using a yeast two-hybrid, UL76 was found to interact with several cellular proteins, including the S5a protein of the ubiquitin-proteasome system (UPS). This interaction involves the conserved region of UL76 (aa 1-190) and the von Willebrand factor type A (VWA) domain of S5a. It induces the accumulation and nuclear relocalization of S5a, thereby sequestering S5a and polyubiquitinated proteins to nuclear aggresomes and inhibiting their proteolytic degradation. The knockdown of S5a using RNA interference led to a reduction in aggresome formation, indicating that UL76-S5a interaction potentially interferes with the import of polyubiquitinated proteins to the 20S proteasome. This is associated with the attenuation of replication compartments, suggesting an association between pUL76, S5a, aggressome formation and replication compartments. Thus, some aggresomes may be UPS compartments associated with replication compartments containing both UL76 and S5a [33].

Finally, by using yeast two-hybrid screening and further confirmation from coimmunoprecipitation, DEV UL54, a potential homolog of HSV-1 ICP27, has been identified as a DEV UL24 partner [41].

## 7. Characteristics of UL24-Deficient Viruses

Examining the outcomes of infection with mutant viruses nullified or mutated in a gene of interest is critical in understanding the importance and function of a given viral gene in the context of various cell models and conditions. This section reviews outcomes of infection with UL24 homolog-deficient or mutated viruses, emphasizing the effects on virus replication and production.

HSV-1 mutants with UL24 containing substitution or frameshift mutations within the conserved amino-terminal domain were first characterized while studying mutations in its overlapping TK gene. A fraction of these mutants exhibited impaired virus growth in cell culture, characterized by small plaques and sometimes syncytia, supporting the importance of the conserved UL24 gene product [29,63,73]. The syncytial phenotype associated with UL24 was particularly evident at elevated temperatures. Its mechanism is still unclear but could be related to the mislocalization of the gB and gD fusogenic viral glycoproteins [65]. Of note, a 2.6-fold higher ratio of nuclear to cytoplasmic particles was evident in cells infected with UL24-deficient HSV-1 compared with wild-type-infected cells, suggesting a defect in nuclear egress of nucleocapsids [62]. A subsequent study employing two HSV-1 UL24-deficient clones that terminate at aa 53 but encode a functional TK revealed reduced replication and a small plaque phenotype. In addition, in a mouse model of corneal infection, these mutants showed an approximately 10-fold decrease in virus titers along with lower levels of viral DNA and LATs expression and a significant decrease in the number of trigeminal ganglia (TG) from which the virus reactivates in an explant assay. This suggests that UL24 is required for both the establishment of latency and reactivation [74]. Formation of syncytial plaques at 390 °C was also evident in HSV-1 UL24 mutant viruses encoding E99A/K101A mutations within the conserved putative endonuclease motif, as well as in G121A UL24 mutant. However, reduced viral yields in vitro, as well as diminished viral titers in the mouse eye and particularly in the TG, without clinical signs, were observed only with E99A/K101A mutant, which also exhibited reduced Nucleolin dispersal similar to UL24-null virus [61,75]. This underscores the dissociation of the temperature-dependent syncytial plaque phenotype of UL24 from other phenotypic characteristics linked to this gene [75]. Similarly, cell monolayers infected with HSV-2 UL24-deficient virus displayed syncytium formation, whereas no syncytia formed upon infection with their corresponding wild-type or the revertant clone. All strains, however, replicated similarly. Additionally, this virus showed reduced virulence after intravaginal inoculation in mice.

Furthermore, although latency was established in the dorsal root ganglia and genital lesions developed upon inoculation of guinea pigs, it was nonlethal, suggesting UL24 as a pathogenicity determinant in these models [76]. Further research used an HSV-2 UL24 mutant to immunize mice, revealing that those immunized through vaginal, footpad, or intramuscular routes were protected from a lethal vaginal challenge of wild-type HSV-2 [77]. In line, TCID50 analysis showed that wild-type PRV titers exceeded those of UL24-null PRV, suggesting a pivotal role for UL24 in PRV propagation [67], whereas PRV encoding UL24 with a nuclear export signal (NES) mutation resulted in a syncytial phenotype, but the viral yield was unaffected [32]. Similarly, the deletion of UL24 in Suid herpesvirus 1 (SuHV-1), an alphaherpesvirus, significantly curtailed replication and spread in Vero cells and reduced virulence in mice, although the UL24-deleted virus retained some degree of lethality [78]. In addition, deletion of UL24 homolog of EHV-1, ORF37, did not affect plaque size and virus replication in different epithelial cells but resulted in impaired virus multiplication in cultivated mouse neural cells in one order of magnitude along with loss of brain and lung pathogenicity [79]. Unlike wild-type-infected animals, mice infected with the ORF37-null virus exhibited no body weight loss, neurological disorders or death. This indicates that ORF37 is required for virus multiplication in neuronal cells and for expression of viral neuropathogenicity in mice, but it is dispensable in certain cell cultures [79]. The replication of the VZV Oka strain ORF35-null showed slower growth kinetics and reduced viral yield in melanoma and Vero cells, with significantly smaller plaques than the control virus [40]. Furthermore, in contrast to melanoma cells infected with the control virus, ORF35-null viruses did not present syncytia. In line with this, the ORF35-null virus inoculated into the SCID-hu mouse model of skin and T-cell xenografts exhibited lower viral titers. Based on comparative growth kinetics and infectious virus yield, however, ORF35 is thought to be more critical for VZV virulence in differentiated human skin cells than in T-cells [40]. Intriguingly, an exploratory endeavor focusing on HSV-1 UL24.5 revealed that a mutant virus designed to substitute the predicted initiation methionine with valine successfully eliminated the expression of the 18-kDa polypeptide. This virus did not exhibit any replication defects in cell culture, although, in a murine model, it was associated with prolonged signs of periocular disease and increased incidence of severe neurological disorders [12].

Based on global transposon mutagenesis in the AD169 hCMV strain and growth kinetics in cultured human fibroblasts, UL76 has been identified as an augmenting viral replication gene product, as its deficiency resulted in small plaques and reduced yields of cell-free virus [80]. A similar study, using the Towne hCMV strain, defined UL76 as essential for viral replication [81]. This discrepancy could stem from differences in strains and the potential impact of deletions, which are not necessarily exactly similar, on adjacent genes in this densely packed genomic region. A later study involving the infection of HFF cells with recombinant Towne strain Bacmids revealed slightly slower growth kinetics of the mutated viruses, which was only evident at low multiplicity of infection and at 5 days post-infection [57]. Notably, UL76 has been shown to have a pivotal role in up-regulating IL-8 during hCMV infection, as evidenced by a notable decrease in secreted IL-8 levels in cells infected with UL76 deletion and UL76 endonuclease motif mutated viruses. Since IL-8 boosts hCMV replication and facilitates effective viral dissemination by neutrophils, it is possible that the effect of UL76 deficiency could be more robust in animal models with an intact immune response rather than in cell cultures [49].

As for gammaherpesviruses, a study involving KSHV ORF20 characterized an ORF20-stop mutant in iSLK-infected cells, showing a notable reduction of viral DNA replication and progeny virions upon lytic reactivation. When, however, ORF20 expression was restored through the introduction of an HA-tagged ORF20 expression plasmid, viral DNA replication and virion production were reinstated, suggesting that the deficiency observed in these cells results from the absence of ORF20 expression, emphasizing the critical role of ORF20 during the lytic cycle [70]. Similarly, our recent study observed a significant reduction in the production of infectious virions in ORF20-null-infected cells compared to wild-type-infected cells despite higher viral DNA levels. Interestingly, we noted accelerated viral gene transcription, early accumulation of lytic proteins, and premature cell death during lytic reactivation in ORF20-null-infected cells, suggesting that ORF20 may play a role in coordinating the lytic cycle and regulating cell death [26]. Notably, the enhancement of KSHV replication by OASL was shown to depend on ORF20 [34]. In contrast, two different MHV-68 clones that do not express ORF20 exhibited wild-type characteristics in cell culture, latency establishment, and viral replication in mice following intranasal infection, other than a noteworthy 4-day delay in viral clearance from the lungs, which may suggest an extension of the lytic replication cycle [82].

## 8. Conclusions and Perspectives

UL24 proteins share conserved homology domains and a putative PD-(D/E)XK endonuclease motif, suggesting a common ancestral origin and potentially conserved functions. The aim of this review is a comprehensive analysis of UL24 functions across different herpesviruses to illuminate both conserved and virus-specific roles. A summary is provided in Table 2.

Generally, UL24 protein expression exhibits late kinetics during viral infection, with some variations among different viruses. They predominantly localize to the nucleus and nucleolus, with certain members showing dynamic distribution patterns during infection. UL24 family members have been implicated in cell-cycle regulation, induction of DNA damage responses, modulation of viral and host gene expression, dispersal of nucleolar proteins, regulation of viral glycoprotein distribution, and evasion of host antiviral responses. They interact with various cellular and viral proteins. Mutant viruses lacking functional UL24 or with mutated PD-(D/E)XK motif often exhibit impaired replication, altered plaque morphology, and reduced pathogenicity in animal models. While the PD-(D/E)XK motif suggests potential endonuclease activity, direct evidence is lacking. Future studies should focus on confirming this activity and its relevance to viral replication and host interactions. Detailed structural analyses of UL24 family members may provide insights into its function and potential as a drug target.

Given the importance of UL24 in viral replication and pathogenesis, it may serve as a target for antiviral therapies. Further investigation of UL24’s role in modulating host immune responses and cellular processes could reveal new aspects of viral pathogenesis that may lead to novel treatment strategies. Characterizing UL24’s protein–protein interactions could uncover new cellular pathways affected by viral infection. Elucidating the precise mechanisms by which UL24 proteins influence cellular processes, such as nucleolar protein dispersal and gene expression regulation, remains an important area for future research. Finally, expanding research into animal models could also provide a better understanding of UL24’s role in viral pathogenesis and immune evasion.

In conclusion, while significant progress has been made in understanding the UL24 protein family, many questions remain unanswered. Continued research into these conserved viral proteins promises valuable insights into herpesvirus biology and will potentially open new avenues for therapeutic intervention.

## Figures and Tables

**Table 1:** UL24 gene cluster in human and selected animal herpesviruses.

Virus	Herpesviridae Subfamily	UL24 Gene	UL24 Flanking Genes	
			Capsid Vertex Component (CVC)	Thymidine Kinase (TK)/Glycoprotein H (gH)
HSV-1 HSV-2	alpha	*UL24*	*UL25*	*UL23 (TK)*
	alpha	*UL24*	*UL25*	*UL23 (TK)*
VZV hCMV	alpha	*ORF35*	*ORF34*	*ORF36 (TK)*
	beta	*UL76*	*UL77*	*UL75 (gH)*
HHV-6A/B HHV-7 EBV KSHV	beta	*U49*	*U50*	*U48 (gH)*
	beta	*U49*	*U50*	*U48 (gH)*
	gamma	*BXRF1*	*BVRF1*	*BXLF1 (TK)*
	gamma	*ORF20*	*ORF19*	*ORF21 (TK)*
MHV-68 TeHV-3	gamma	*ORF20*	*ORF19*	*ORF21 (TK)*
	alpha	*UL24*	*UL25*	*UL23 (TK)*
DEV	alpha	*UL24*	*UL25*	*UL23 (TK)*
PRV	alpha	*UL24*	*UL25*	*UL23 (TK)*
BHV-1	alpha	*UL24*	*UL25*	*UL23 (TK)*
EHV-1	alpha	*ORF37*	*ORF36*	*ORF38 (TK)*

**Table 2:** Major characteristics of members of the UL24 family.

HSV-1-UL24	PRV-UL24	HSV-2-UL24	VZV-ORF35	EBV-BXRF1	hCMV-UL76	KSHV-ORF20	MHV-68-ORF20
**Expression Kinetics**							
Leaky-late [7,8,12]	Late [10]	Late [9]	Unknown	Unknown	Late [13,14,15]	Late [24,25,28]	Early-late [27]
**Endonuclease Motif**							
+	+	+	+	+	+	+	+
**Cellular Localization**							
Nuclear [11] Cytoplamic and nuclear [37] Golgi, nuclear, and nucleolar [31,38,61]	Nucleus [39]	Cytoplasmic, nuclear and nucleolar [9]	Nuclear [40]	Nucleolar [44]	Nuclear and nucleolar [13,33,38]	Nuclear and nucleolar [34,43,70]	Nuclear [45]
**Function**							
Induction of cell cycle arrest and apoptosis [46] Modulation of gene expression [51] Evasion of antiviral response [52] Suppression of the NF-κB signaling pathway [52] Alterations of cellular distribution of viral envelope fusion glycoprotein [64] Redistribution of selected nucleolar proteins [31,37,60,61,62]	Evasion of antiviral response [52,56,66,67] Suppression of the NF-κB signaling pathway [53]	Unknown	Unknown	Unknown	Induction of cell cycle arrest and apoptosis [46] Induction of DNA damage response [46,47,48,49] Modulation of gene expression [13,49,57] Evasion of antiviral response [49] Activation of the NF-κB signaling pathway [49]	Induction of cell cycle arrest and apoptosis [46]	Induction of cell cycle arrest and apoptosis [45]
**Protein–Protein Interaction**							
NF-κB p65 and p50 subunits [52]	NF-κB p65-subunit [39] IRF-7 [53]	Unknown	Unknown	Unknown	Viral pUL31 [71] S5a [33] PARP-1 [50]	CCDC86 [34] OASL [34] KSHV ORF59 [70] Ribosomal 40S and 60S proteins [34,70]	
